# Silencing of lncRNA MIR497HG via CRISPR/Cas13d Induces Bladder Cancer Progression Through Promoting the Crosstalk Between Hippo/Yap and TGF-β/Smad Signaling

**DOI:** 10.3389/fmolb.2020.616768

**Published:** 2020-12-09

**Authors:** Changshui Zhuang, Ying Liu, Shengqiang Fu, Chaobo Yuan, Jingwen Luo, Xueting Huang, Weifeng Yang, Wuwei Xie, Chengle Zhuang

**Affiliations:** ^1^Department of Urology, Union Shenzhen Hospital, Huazhong University of Science and Technology, Shenzhen, China; ^2^Shenzhen People’s Hospital, The First Affiliated Hospital of Southern University of Science and Technology, The Second Clinical Medical College of Jinan University, Shenzhen, China; ^3^Department of Urology, The First Affiliated Hospital of Nanchang University, Nanchang, China; ^4^Emergency Department, Union Shenzhen Hospital, Huazhong University of Science and Technology, Shenzhen, China; ^6^Shenzhen Yantian District People’s Hospital, Shenzhen, China; ^5^Department of Thoracic Surgery, Union Shenzhen Hospital, Huazhong University of Science and Technology, Shenzhen, China; ^7^Department of Urology, Peking University Shenzhen Hospital, Shenzhen, China

**Keywords:** bladder cancer, MIR497HG, CRISPR/Cas13d, Yap, TGF-β

## Abstract

A subset of long non-coding RNAs (lncRNAs), categorized as miRNA-host gene lncRNAs (lnc-miRHGs), is processed to produce miRNAs and involved in cancer progression. This work aimed to investigate the influences and the molecular mechanisms of lnc-miRHGs MIR497HG in bladder cancer (BCa). The miR-497 and miR-195 were derived from MIR497HG. We identified that lnc-miRHG MIR497HG and two harbored miRNAs, miR-497 and miR-195, were downregulated in BCa by analyzing The Cancer Genome Atlas and our dataset. Silencing of MIR497HG by CRISPR/Cas13d in BCa cell line 5637 promoted cell growth, migration, and invasion *in vitro*. Conversely, overexpression of MIR497HG suppressed cell progression in BCa cell line T24. MiR-497/miR-195 mimics rescued significantly the oncogenic roles of knockdown of MIR497HG by CRISPR/Cas13d in BCa. Mechanistically, miR-497 and miR-195 co-ordinately suppressed multiple key components in Hippo/Yap and transforming growth factor β signaling and particularly attenuated the interaction between Yap and Smad3. In addition, E2F4 was proven to be critical for silencing MIR497HG transcription in BCa cells. In short, we propose for the first time to reveal the function and mechanisms of MIR497HG in BCa. Blocking the pathological process may be a potential strategy for the treatment of BCa.

## Introduction

Bladder cancer (BCa) is one of the most prevalent epithelial malignancies worldwide (Sanli et al., 2017). Most of the diagnosed patients have non–muscle-invasive BCa (MIBC) confined to the mucosa or lamina propria, and approximately 25% have MIBC that invades the detrusor muscle (Burger et al., 2013; Robertson et al., 2018). MIBC is more aggressive and have a worse prognosis (Felsenstein and Theodorescu, 2018; Robertson et al., 2018). Existing therapies for MIBC have not changed mortality rates over the past years (Felsenstein and Theodorescu, 2018). Genome and transcriptome profiling studies have revealed considerable differences in the molecular and genetic features of BCa cells, such as mutations, copy number, and gene epigenetic alterations, determining tumor heterogeneity and therapeutic resistance (Guo G. et al., 2013; Robertson et al., 2018; Kamoun et al., 2020). However, non-genetic or epigenetic mechanisms in BCa remain elusive.

Non-coding RNAs (ncRNAs), particularly long non-coding RNAs (lncRNAs, >200 nt) and microRNAs (miRNAs, 20–22 nt), are critical for epigenetic regulation (Spitale et al., 2011; Ameres and Zamore, 2013). Recent studies have reported that certain lncRNAs are referred to as miRNA-host gene and acquire functionality by serving as the precursor to microRNAs capable of regulatory role (Rodriguez et al., 2004; Cai and Cullen, 2007; Dhir et al., 2015). For example, MIR100HG-derived miR-100 and miR-125b induce chemotherapy resistance via augmenting Wnt pathway (Lu et al., 2017). MiR-675 derives from lncRNA-H19 and inhibits cell proliferation in response to cellular stress or oncogenic signals (Keniry et al., 2012). MiR-17∼92 cluster miRNAs encoded by MIR17HG downregulate transforming growth factor β (TGF-β) and STAT3 signaling in Feingold syndrome mouse models (Mirzamohammadi et al., 2018). LncRNA MIR497HG and derived miR-497∼195 cluster are downregulated in BCa (Itesako et al., 2014; Eissa et al., 2019). Furthermore, the expression of miR-195 and miR-497 is inhibited, and they function as tumor suppressors in various types of human cancer including breast cancer (Li et al., 2011), lung cancer (Chae et al., 2019), colorectal cancer (Guo S. T. et al., 2013), and hepatocellular carcinoma (Furuta et al., 2013). However, the impact of these lncRNAs or derived miRNAs in BCa progression is largely unknown.

The function of MIR497HG was verified via forward and reverse validation in BCa. To inhibit the expression of MIR497HG, we design siRNAs targeting MIR497HG while the expression of MIR497HG was not changed after transfection of siRNAs in our work. Other method to restrain MIR497HG expression is carried out in this study. New discovered type VI CRISPR system is used to suppress the expression of MIR497HG. A single protein effector (Cas13) can cleave a specific RNA with a single RNA (crRNA) in this system (Smargon et al., 2017). There are four subtypes (A–D) in type VI systems (Wang et al., 2019). Type VI-D CRISPR effectors, known as RfxCas13d, were recently discovered and used for RNA depletion with high efficiency and specificity in mammalian cells, including cancer (Abudayyeh et al., 2017; Granados-Riveron and Aquino-Jarquin, 2018).

Many signaling pathways such as JAK-STAT, nuclear factor κB, mTOR, and mitogen-activated protein kinase have been reported to affect the survival of BCa (Abbosh et al., 2015). Evolutionarily conserved Hippo pathway has been shown to paly critical roles during BCa progression and tumorigenesis (Xia et al., 2018). The Hippo pathway regulates cell proliferation and migration via the downstream effector Yes-associated protein (Yap; Mo et al., 2014). Yap is highly expressed and acts as oncogenes in BCa (Liu et al., 2013; Ciamporcero et al., 2016). TGF-β serine/threonine kinase complex binds to TGF-β receptors and further activates different downstream substrates and regulatory proteins, mainly the SMAD, inducing transcriptions of different target genes involved in cell proliferation and differentiation (Massagué, 2012). Besides, activation of the TGF-β/Smad signaling pathway often co-ordinates with Hippo/Yap pathway in human cancer, including BCa (Dong et al., 2019).

Here we report that MIR497HG plays antitumorigenic roles in BCa. We found that miR-497, miR-195, and their host gene MIR497HG were low expressed in BCa tissues and cell lines. MiR-497 and miR-195 synergistically targeted Yap and SMAD3 and their downstream genes and decreased the formation of a Yap-SMAD complex (Luo, 2017). We also identified that E2F4 is a critical transcriptional suppressor of MIR497HG. Our findings uncover an ncRNAs-mediated epigenetic mechanism to block the crosstalk between Hippo/Yap and TGFβ/Smad signaling. It may contribute to search for effective personalized targeted therapies to treat BCa.

## Materials and Methods

### Cell Culture

SV-HUC-1 (normal human immortalized urothelial cell line) and the human BCa cell lines T24, 5637, RT4, UM-UC-3, SW780, and TCCSUP were purchased from the American Type Culture Collection (ATCC, Manassas, VA, United States). T24, 5637, and SW780 were cultured in RPMI-1640 medium. UM-UC-3 and TCCSUP were cultured in DMEM. RT4 was maintained in McCoy’s 5a, and SV-HUC-1 was cultured in F-12K medium. Ten percent fetal bovine serum (Biological Industries, Beit Haemek, Israel) and 1% penicillin/streptomycin (GIBCO, Gaithersburg, MD, United States) were added to obtain complete growth medium.

### BCa Specimens

All BCa samples were collected from the Peking University Shenzhen Hospital and Shenzhen University Nanshan Hospital. All patients signed written informed consent, and this study was approved by the Ethics Committee of Shenzhen University Nanshan Hospital. The pathological status of the specimens was provided by the board-certified pathologist.

### Western Blot

Protein extracts were prepared in RIPA lysis buffer (#P0013B; Beyotime), supplemented with phenylmethylsulfonyl fluoride (PMSF; protease inhibitor and phosphatase inhibitor cocktail). Protein concentrations were determined using bicinchoninic acid kit (Sigma–Aldrich) according to the manufacturer’s protocol. sodium dodecyl sulfate–polyacrylamide gel electrophoresis was used to resolve cell lysates and transferred onto polyvinylidene fluoride membranes. Membranes were incubated for 1 h with non-fat milk in Tris Buffered Saline Tween (TBST) buffer and incubated overnight at 4°C with primary antibodies and required secondary antibodies conjugated to horseradish peroxidase and developed by chemiluminescent substrates.

### Reverse Transcription–Quantitative Polymerase Chain Reaction and Chromatin Immunoprecipitation

TRIzol^TM^ Reagent (Invitrogen) was utilized to extract total RNA, and RNA was purified using RNeasy Mini Columns (Qiagen), according to the manufacturer’s protocol. For mRNA and lncRNA MIR497HG detection, cDNA was generated using SureScript^TM^ First-Strand cDNA Synthesis Kit (GeneCopoeia). Quantitative polymerase chain reaction (qPCR) was then performed using the SYBR Green qPCR MasterMix (Takara). To analyze the expression level of miR-195 and miR-497, cDNA was synthesized by a mir-X miRNA First-Strand Synthesis Kit (Takara, Dalian, China). MiRNA expression was used for mir-X miRNA quantitative reverse transcription (qRT)–PCR SYBR kit (Toyobo, Osaka, Japan) according to the manufacturer’s instructions, with U6 as the control.

### Chromatin Immunoprecipitation

Chromatin immunoprecipitation (ChIP) assays were performed according to manufacturer’s instructions using Cell Signaling Immunoprecipitation Kit (#9002). Briefly, 5637 cells were fixed with paraformaldehyde and lysed in buffer A supplemented with DTT. Protease inhibitor cocktail (PIC) and PMSF were maintained on ice for 10 min. Nuclei were pelleted and resuspended in buffer B + DTT. DNA was digested with 0.5 μL micrococcal nuclease. Nuclei were again pelleted, resuspended in ChIP buffer with PIC and PMSF, and incubated at 4°C. The lysates were clarified by centrifugation, and ChIP was carried out by incubating the sample with rabbit immunoglobulin G or E2F4 antibody (Abcam), followed by immobilization on protein A/G-agarose beads (Life Technologies). The chromatin was eluted from antibody/protein G agarose beads, cross-links were reversed, and DNA was purified using spin columns. The qPCR was performed using MIR497HG promoter or GAPDH primers. Ct values were normalized to input DNA. The detailed primer sequences for RT-qPCR and ChIP assays are listed in Supplementary Table 1.

### Cell Counting Kit-8 (CCK-8) Assay

Cells were cultured in 96-well plates at a concentration of 3 × 10^3^ cells per well. After treatment, 10 μL CCK-8 reagent (Dojindo, Kumamoto, Kyushu, Japan) was added to each well to react for 0.5 h. The absorbance was measured at 450 nm using a microplate reader.

### Colony Formation Assay

One thousand cells cells/well were plated onto six-well plates, which were incubated at 37°C and 5% CO_2_ until colonies were formed. After 10 to 15 days, colonies were fixed using 0.05% crystal violet in 4% paraformaldehyde and counted using ImageJ program.

### Cell Migration Assay

Cells were seeded in six-well plate (5 × 10^5^/per well) and incubated at 37°C in a humidified incubator containing 5% CO_2_ to get 100% confluence before transfection. A clear line was produced with scratching using a sterile 200-μL pipette tip. Images were taken from each well quickly. After 24 h, pictures were taken again with the help of a digital camera system. The time of 0 and 24 h of migration distance was calculated, and assays were performed at least three times.

### Cell Invasion Assay

Cells were transfected with siRNAs/plasmids for 48 h and then trypsinized and resuspended in serum-free medium. Cells (1 × 10^5^) were then added to the upper chambers of the Transwell inserts (Millicell; Merck KGaA) and allowed to migrate toward the bottom of the chambers. After 24 h, the remaining cells in the upper chamber were removed, and at room temperature, cells on the underside were fixed in 4% paraformaldehyde for 30 min and stained with 0.1% crystal violet for 30 min and captured using an Olympus-type light microscope sz30. Quantification of the migrated cells was performed by counting cell numbers.

### Plasmids, Lentiviral Production, and Transfection

Yes-associated protein1 and SMAD3 coding sequence was cloned into pCMV-HA-N expression vector (*Sal*I/*Bgl*II and *Kpn*I/*Not*I). Cas13d vector was obtained from Addgene 109049, and crRNA was designed according to the previous study (Abudayyeh et al., 2017). The crRNA sequence was designed according to the website, https://cas13design.nygenome.org/, and the top three crRNA sequences were chosen for this study (AAGAGCAAAATTTAGGGTGCA TC, GA GCAAAATTTAGGGTGCATCCC, and AGAGCAAAATT TAGGGTGCAT CC). The primer sequences are presented in Supplementary Table 1. pLemiR control, pLemiR-195, or pLemiR-497 plasmid was packaged with pMDL, VSVG, and pRSV-Rev into HEK-293T cells. To establish stable cell lines, the concentrated lentivirus was directly added into cancer cells and incubated at 37°C for 48 h before they were washed out with phosphate-buffered saline. Finally, cells were selected with 2.5 mg/mL puromycin for 4 days. The 3′ UTR fragments of Yap1, SMAD3, CCND1, and BIRC5 containing the wild-type or mutant miR-497∼195 cluster putative target sites were directly synthesized from GeneCreate (Wuhan, China) and cloned downstream of the Renilla luciferase cassette in psiCHECK-2 (Promega). The connective tissue growth factor (CTGF)–luc plasmids were generated as described previously (Zhao et al., 2008). The promoter fragments of human MIR497HG were directly synthesized from GeneCreate (Wuhan, China) and cloned into pGL3-Basic vector (Promega). A site-directed mutagenesis kit (Thermo Fisher Scientific) was used to mutate the miR-497∼195 cluster or E2F4-binding sites of these vectors. The primer sequences used for the site-directed mutagenesis are provided in Supplementary Table 1. All sequences were confirmed by sequencing.

The miRNA inhibitors and mimics of miR-ctrl, miR-195, and miR-497 were obtained from Ribobio (Guangzhou, China). Lipofectamine 3000 (Invitrogen, Carlsbad, CA, United States) was used to transfect 50 nM of mimic or inhibitor of miR-ctrl, miR-195, and miR-497 into indicated cancer cells according to the manufacturer’s protocol. The expression levels of miR-195 and miR-497 were quantified after 48-h transfection.

### Luciferase Reporter Assay

miR-497 or miR-195 mimic or control mimic (Ambion) and indicated psiCHECK-2-3′ UTR wild-type or mutant plasmids were co-transfected into 5637 cells cultured in 24-well plates using Lipofectamine 3000 (Thermo Fisher Scientific). Renilla and firefly luciferase activities were tested with the dual-luciferase reporter assay system (Promega, Madison, WI, United States) according to the manufacturer’s manual.

In order to measure promoter activities, the MIR497HG promoter fragment sequence was inserted into pGL3-basic plasmid, named pGL3-MIR497HG. Then pcDNA3.1-E2F4 expression plasmid or empty vector control and pGL3-MIR497HG or pGL3-MIR497HG-mut were co-transfected into 5637 cells cultured in 24-well plates using Lipofectamine 3000 (Thermo Fisher Scientific). The firefly and Renilla luciferase activity was measured after 48 h with the dual-luciferase reporter assay system (Promega). Firefly luciferase activity was normalized to Renilla activity.

### Statistical Analysis

Statistical analysis was performed by the SPSS 21 (SPSS Inc., Chicago, IL, United States). Two-tailed unpaired or paired Student *t* test, analysis of variance (ANOVA; Dunnett or least significant difference *post hoc* test), and Pearson correlation coefficients were used according to the type of experiment. The statistical significance between data sets was expressed as *P* values, and *P* < 0.05 was considered significant, ^∗^*P* < 0.05, ^∗∗^*P* < 0.01, and ^∗∗∗^*P* < 0.001.

## Results

### MIR497HG and MIR497HG-Derived miR-497 and miR-195 Were Downregulated in BCa

**FIGURE 1:**
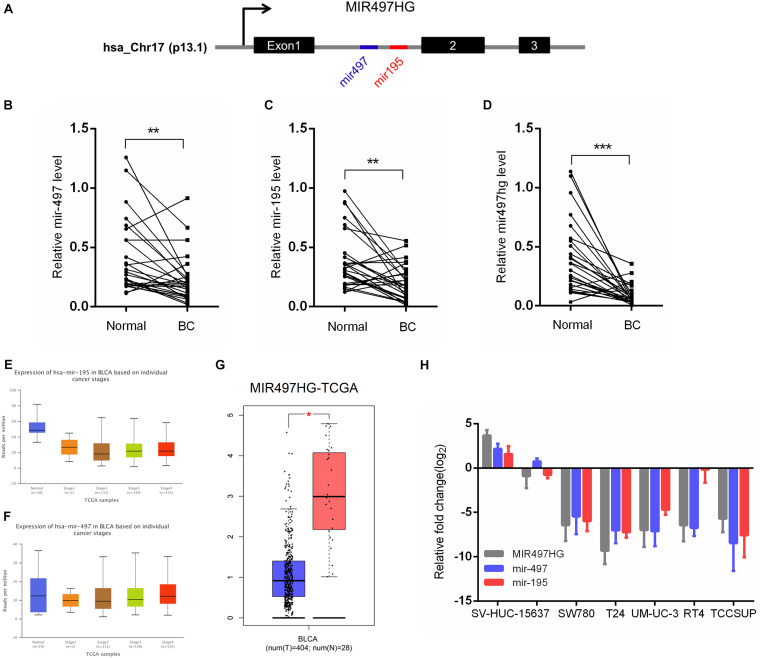
MIR497HG and embedded miR-497 and miR-195 were downregulated in bladder cancer. **(A)** Genomic representation of MIR497HG, host gene of the miR-497/195 cluster, was shown on human chromosome 17. **(B–D)** The expression of MIR497HG and miR-497/195 cluster analyzed by qRT-PCR in 27 paired BCa tissues and adjacent normal tissues. **P < 0.01 and ***P < 0.001 by paired-samples *t* test. **(E–G)** TGCA bladder cancer datasets were analyzed for MIR497HG and miR-497/195 cluster expression. *P < 0.05. **(H)** The qRT-PCR analysis of MIR497HG, miR-497, and miR-195 expression levels among a panel of 7 BCa cell lines. GAPDH or U6 snRNA served as the internal control.

MIR497HG is the host gene of the miR-497∼195 cluster on chromosome 17 (Figure 1A). We first examined the expression of MIR497HG, miR-497, and miR-195. The RT-qPCR analysis confirmed the downregulation of endogenous MIR497HG, miR-497, and miR-195 expression in BCa tissues compared with normal adjacent tissues (Figures 1B–D). Furthermore, we analyzed The Cancer Genome Atlas (TCGA) bladder urothelial carcinoma RNA sequencing data and revealed that the expression levels of miR-497 and miR-195 were inhibited in various stage of BCa (Figures 1E,F), and these data also showed that MIR497HG was downregulated in BCa (Figure 1G). Similarly, the expression levels of MIR497HG, miR-195, and miR-497 were repressed in different BCa cell lines including 5637, T24, UMUC-3, SW780, RT4, and TCCSUP. Thus, MIR497HG, miR-497, and miR-195 were downregulated in both human BCa tissues and cell lines (Figure 1H). These data suggest that MIR497HG, miR-497, and miR-195 may be involved in the progression of BCa.

### MIR497HG Suppressed BCa Cell Growth, Migration, and Invasion *in vitro*

**FIGURE 2:**
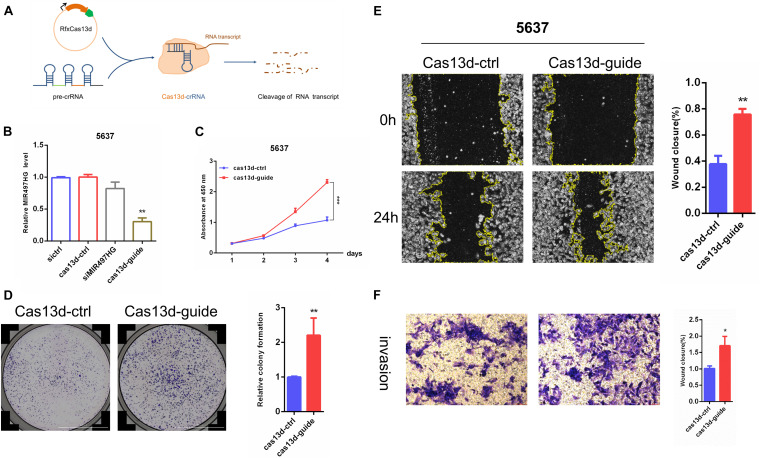
MIR497HG suppresses BCa cell growth, migration, and invasion *in vitro*. **(A)** The schematic of CRISPR/Cas13d system. **(B)** Knockdown of MIR497HG with Cas13d compared with corresponding RNAi. **(C)** The CCK-8 assays showed that silencing of MIR497HG promotes BCa cell proliferation in 5637 cell line. **(D)** Clonogenic assay was performed to measure the capacity of foci formation in 5637 cells stably expressing indicated plasmids. **(E)** Wound healing assay of 5637 cells stably expressing indicated plasmids. **(F)** Representative images of Transwell invasion assay for MIR497HG-silencing 5637 cells. Cell number was counted in six randomly captured pictures. Invasive ability was normalized to control. Data are shown as mean ± SD. *n* = 3 for technical replicates. *P < 0.05, **P < 0.01, and ***P < 0.001. *P* values are calculated by Student *t* test.

Forward and reverse validation of the function of MIR497HG was verified in BCa cells. The expression level of MIR497HG was not changed after transfection of three different siRNAs targeting MIR497HG (Supplementary Figure 1). Thus, CRISPR/Cas13d system (crRNA and Cas13d) was used in this study, and the schematic of this system is shown in Figure 2A. Although the expression level of MIR497HG was decreased significantly in both 5637 and T24 cells, it was higher in 5637 than that in T24. Thus, the expression of MIR497HG was inhibited significantly using CRISPR/Cas13d targeting MIR497HG in BCa 5637 cells (Figure 2B). CCK-8 assays and colony formation assays showed that inhibition of MIR497HG via CRISPR/Cas13d significantly promoted cell proliferation, migration, and invasion in 5637 (Figures 2C–F). Conversely, overexpression of MIR497HG in BCa T24 cells restrained significantly cell growth (Supplementary Figures 2A–C), scratch wound healing (Supplementary Figures 2D,E), and invasion abilities (Supplementary Figures 2F,G) compared with vector control. However, MIR497HG has no effects on cell apoptosis in BCa cells (data was not shown). Thus, MIR497HG was regarded as a tumor suppressor gene in BCa.

### MiR-497 and miR-195 Rescued the Enhancing Effects of MIR497HG Inhibition via CRISPR/Cas13d on BCa Progression

**FIGURE 3:**
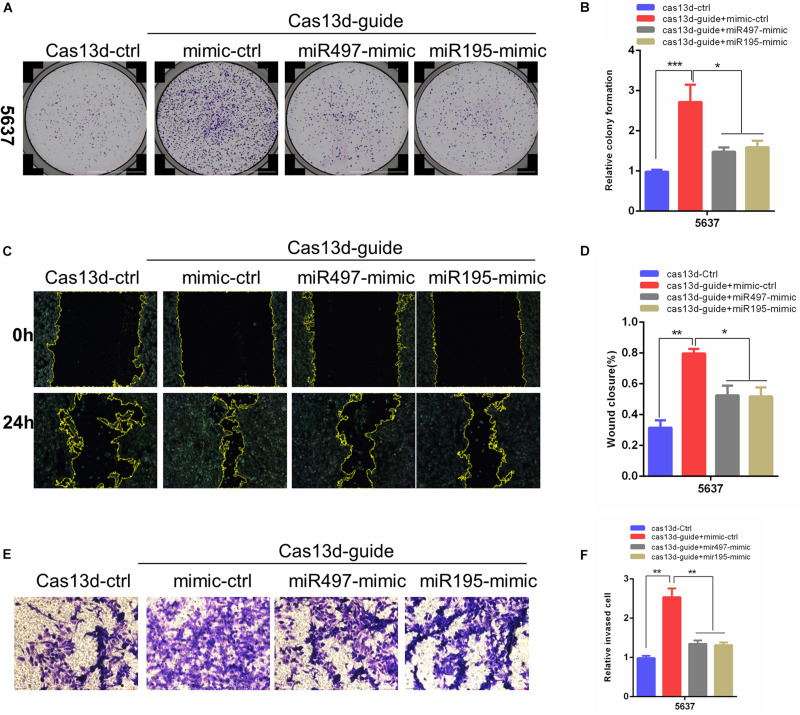
MiR-497 and miR-195 rescued the enhancing effects of MIR497HG inhibition via CRISPR/Cas13d on BCa progression. **(A, B)** Clonogenic assay showed that miR-497 or miR-195 mimics reversed partial Cas13d-mediated silenced MIR497HG enhancing growth of BCa cell. Wound healing assays **(C, D)** and Transwell assays **(E, F)** indicated that miR-497 or miR-195 mimics rescued the enhancing function of Cas13d-mediated MIR497HG knockdown on migratory and invasive activity of 5637 cells. Data are shown as mean ± SD. *n* = 3 for technical replicates. *P < 0.05, **P < 0.01, and ***P < 0.001. *P* values are calculated by Student *t* test.

Considering that a major role of miRNA-host gene lncRNAs (lnc-miRHGs) depends on their derived miRNAs (Augoff et al., 2012; Keniry et al., 2012; Lu et al., 2017; Wu et al., 2017), we test whether the phenotypes associated with MIR497HG are mediated by miR-497 and miR-195 in BCa cell lines. miR-497 and miR-195 mimics were transiently transfected into 5637 cells after MIR497HG inhibition through CRISPR/Cas13d tool. The most significant suppressive effect was detected using crRNA sequence 1, and we picked these sequences for further study (data was not presented). As shown in Figure 3, miR-497 or miR-195 mimics partially reversed the promotion of cell proliferation (Figures 3A,B), migration (Figures 3C,D), and invasion (Figures 3E,F) induced by MIR497HG suppression. Collectively, these data suggested that miR-497 and miR-195 are indispensable in the biological function of MIR497HG in BCa.

### MiR-497 and miR-195 Directly Suppressed Multiple Key Components in Hippo/Yap and TGF-β Signaling

**FIGURE 4:**
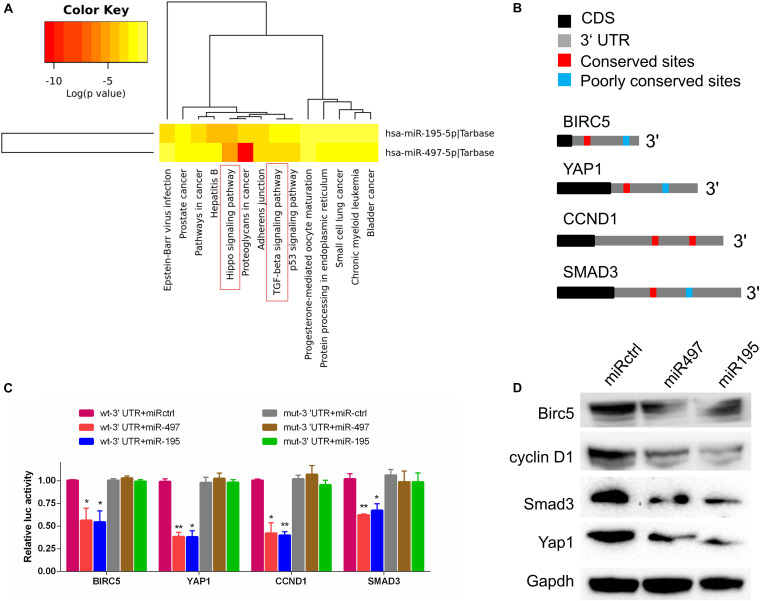
MiR-497 and miR-195 directly suppressed multiple key components in Hippo/Yap and TGF-β signaling. **(A)** The Kyoto Encyclopedia of Genes and Genomes analysis of miR-497 and miR-195 target genes. **(B)** Predicted miR-497 and miR-195 binding conversed (red) and poorly conversed (blue) sites in 3′ UTRs of Birc5, Yap1, CCND1, and Smad3. CDS, coding sequence. **(C)** Luciferase reporter assay of candidates predicted to be regulated by miR-497 or miR-195. Renilla luciferase activity was normalized to firefly activity. *n* = 3 independent experiments. *P < 0.01 and **P < 0.01 by Student *t* test. **(D)** Western blot analysis of Birc5, cyclin D1, Smad3, and Yap1 in the indicated cells. Gapdh served as the loading control. Representative of three independent experiments.

To explore the molecular pathways of miR-195 and miR-497 in BCa cells, we performed *in silico* analyses using mirPath v.3 and TargetScan. Based on the Kyoto Encyclopedia of Genes and Genomes pathway enrichment analysis, 14 pathways were enriched in miR-195/497 cluster putative targets (Figure 4A and Supplementary Table 2). Then, we focused on genes predicted as miR-195/497 cluster targets and involved in Hippo signaling and TGF-β pathway. Analyses of TargetScan database and previous studies (Li et al., 2011; Itesako et al., 2014; Jafarzadeh et al., 2016; Zhang L. et al., 2016) revealed that 3′ UTRs of Yap, SMAD3, CCND1, and BIRC5 contained at least one conserved binding site for miR-497∼195 cluster (Figure 4B). These genes are the key components in the Hippo signaling and TGF-β pathway. Next, luciferase reporter assays confirmed that miR-497 and miR-195 directly target the 3′ UTR of these candidates (Figure 4C). The RT-qPCR and Western blot analysis showed that miR-497 or miR-195 mimics dramatically inhibited Yap, Smad3, Ccnd1, and Birc5 expression in BCa 5637 cells (Figure 4D and Supplementary Figure 3).

### MiR-497 and miR-195 Co-ordinately Attenuated Yap- and Smad3-Dependent Transcriptional Activity

**FIGURE 5:**
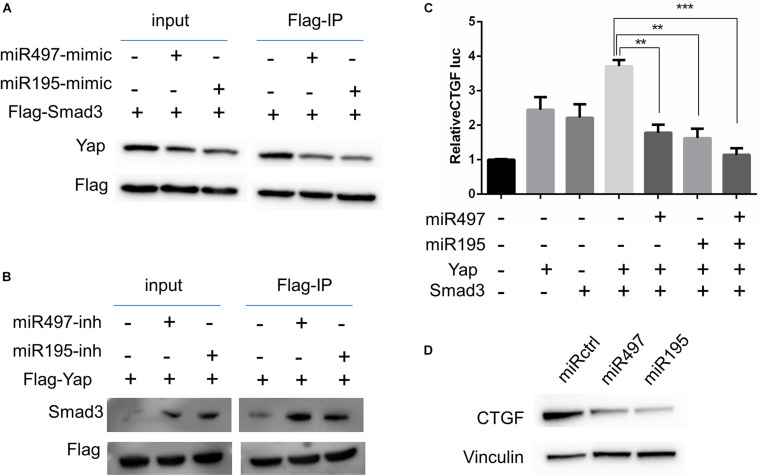
MiR-497 and miR-195 co-ordinately attenuated Yap and Smad3 dependent transcriptional activity. **(A, B)** 5637 cells were transfected with expression vectors as indicated. Cell lysates were subjected to immunoprecipitation (IP) with an anti-Flag antibody and subsequent WB with indicated antibodies. **(C)** CTGF-luciferase reporter was co-transfected with other plasmids into 5637 cells as indicated for luciferase assay. Results are expressed as mean ± SD. **P < 0.01 and ***P < 0.001. **(D)** Western blot analysis of CTGF in the cells transfected with miR-497 and miR-195 mimics.

Previous studies suggest that Yap–Smad3 interaction is essential for the crosstalk between Hippo signaling and TGF-β pathway (Varelas et al., 2008; Fujii et al., 2012; Luo, 2017). To examine whether miR-497∼195 clusters affect the formation of Yap–Smad complex, we performed immunoprecipitations to detect Yap–Smad3 interaction in 5637 cell lines. Our results showed that the interaction between Yap and Smad3 was inhibited/enhanced via miR-497 or miR-195 mimic/inhibitor treatment, respectively, (Figures 5A,B). Interaction between Yap and Smad3 is required to positively regulate transcriptional activity of their common downstream genes including CTGF, a critically oncogenic target (Fujii et al., 2012). Next, CTGF luciferase assays showed that miR-497∼195 clusters blocked CTGF promoter transcriptional activity mediated by Yap and Smad3 (Figure 5C). CTGF protein expression was decreased with miR-497 or miR-195 overexpression (Figure 5D). These data suggest that miR-497 and miR-195 synergistically repress Yap- and Smad3-mediated transcriptional activity of CTGF.

### E2F4 Transcriptionally Repressed MIR497HG Expression in BCa Cells

**FIGURE 6:**
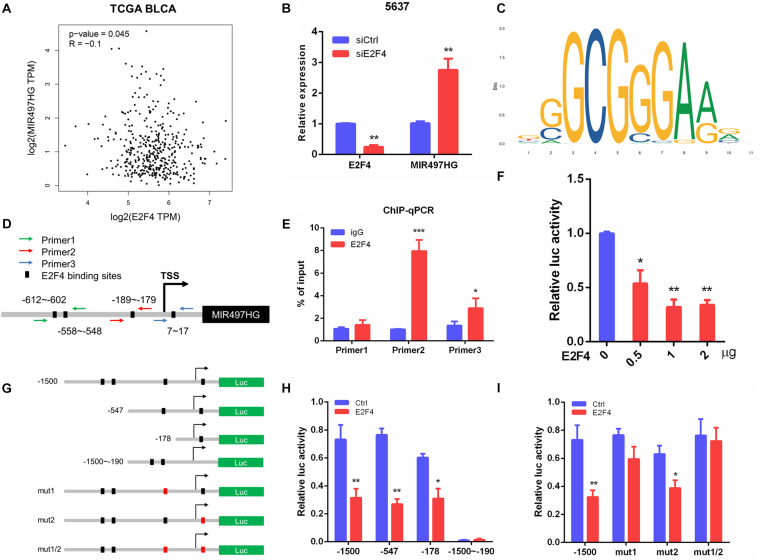
E2F4 transcriptionally repressed MIR497HG expression in BCa cells. **(A)** The correlation between E2F4 gene expression and MIR497HG level in TCGA datasets. **(B)** 5637 cells expressing either E2F4 or non-silencing control siRNA were analyzed for E2F4 (left) or MIR497HG (right) mRNA expression using qRT-PCR. **(C)** E2F4-binding motif. **(D)** E2F4 binding in the MIR497HG promoter and primer designation. **(E)** Chromatin immunoprecipitation was performed in 5637 cells with either IgG or E2F4 and subsequently subjected to qPCR analysis with the indicated primers. Student *t* test, *P < 0.05 and ***P < 0.001. **(F)** HIF-1a promoter activity was determined in 5637 with E2F4 overexpression. One-way ANOVA with Dunnett posttest, *P < 0.05 and **P < 0.01. **(G)** A schematic representation of deletion or mutation constructs spanning the −1,500 to + 200 region of the MIR497HG promoter. **(H, I)** The luciferase vector pGL3 driven by either wild-type, deletion, or mutant MIR497 promoter was transfected in 5637 cells, and luciferase activity was measured. *n* = 3 independent experiments. *P < 0.05 and **P < 0.01 by Student *t* test.

**FIGURE 7:**
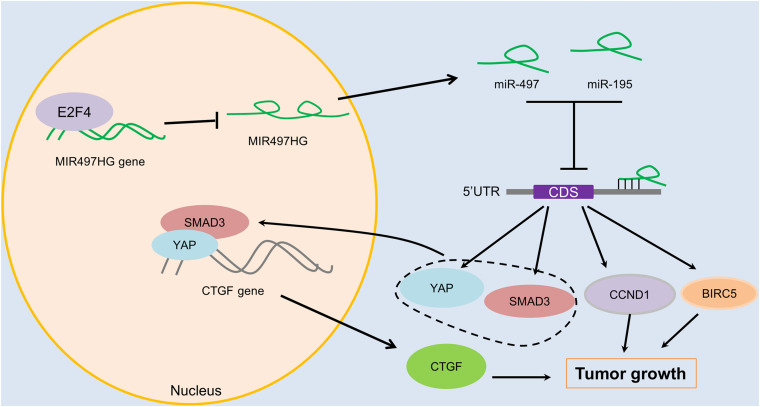
The schematic diagram of mechanism of lncRNA MIR497HG in BCa. E2F4 bound to the promoter of MIR497HG to act as inhibitory transcription factors to suppress the expression of MIR497HG. miR-497 and miR-195 were derived from MIR497HG, and they suppressed the crosstalk of Yap and SMAD3, which were key components in Hippo/Yap and TGF-β/Smad signaling. Then, the downstream of Yap/SMAD3 complex, oncogenic CTGF, was restrained to curb cell progression of BCa.

To determine the mechanism of downregulation of miR-497∼195 clusters in BCa, we analyzed the host gene MIR497HG promoter sequence using MethPrimer and JASPAR. First, no CpG island exists in the promoter region of MIR497HG predicted by MethPrimer. It suggested that MIR497HG expression might not be repressed by promoter methylation. Then we identified conserved DNA-binding sites for fork head/winged helix transcription factors. Among these transcription factors, we focused on E2F transcription factor 4 (E2F4), which was negatively correlated with MIR497HG expression (Figure 6A) and had relatively high scores (Supplementary Table 3). Therefore, we individually knocked down E2F4 or E2F6 (another high score transcription factor) in 5637 cell line using siRNA and analyzed the effect of these knockdowns on MIR497HG expression. Silencing of E2F4 significantly promoted MIR497HG expression (Figure 6B), except knockdown of E2F6 (Supplementary Figure 4). It inspired us to determine whether the transcription factor E2F4 directly targets MIR497HG. Thus, we first utilized the Ensembl and JASPAR to identify forkhead/winged helix motif (GGCGGGAA) in the MIR497HG 1.5-kb promoter region and found four potential binding sites (Figures 6C,D). Next, real-time PCR after ChIP confirmed that E2F4 was significantly enriched at the MIR497HG promoter (Figure 6E). We further confirmed that E2F4 directly regulates MIR497HG expression using luciferase reporter assays. E2F4 overexpression significantly decreased the activity of the MIR497HG promoter (Figure 6F). Sequential mutations and deletions of four binding sites revealed that forkhead/winged helix-binding site “−189∼−179” was the major site for E2F4 repressing MIR497HG transcriptional activity (Figures 6G–I). Altogether, these data clearly demonstrate that E2F4 inhibited MIR497HG transcription by directly binding to its promoter in BCa cells. The schematic diagram of mechanism of lncRNA MIR497HG is shown in Figure 7. E2F4 bound to the promoter of MIR497HG to inhibit the expression of MIR497HG. miR-497 and miR-195 were derived from MIR497HG and suppressed the crosstalk of Yap and SMAD3, which were key components in Hippo/Yap and TGF-β/Smad signaling. Then, the downstream of Yap/SMAD3 complex, oncogenic CTGF, was restrained to curb cell proliferation of BCa.

## Discussion

MIR497HG is a host gene of the miR-497 and miR-195 embedded in its first intron. MIR497HG was significantly downregulated and might serve as a potential diagnostic marker in BCa (Eissa et al., 2019). MiR-495 and miR-195 were also reduced in BCa and inhibited cancer cell progression (Han et al., 2011; Itesako et al., 2014; Zhang Y. et al., 2016). Consistent with these findings, we confirmed that concomitant low expression of MIR497HG, miR-497, and miR-195 occurred in BCa and that miR-497 and miR-195 co-ordinately play critical antioncogenic roles in BCa cells. *In vitro*, we found that inhibition/overexpression of MIR497HG significantly promotes/inhibits the viability and proliferation of BCa cells. No properly designed siRNAs may be the possible reason for the ineffectiveness of the siRNAs targeting MIR497HG. CRISPR/Cas13d was used to knock down the expression of MIR497HG. Compared with other type VI effectors, the size of Cas13d is smaller and has been verified as effective tools for RNA targeting and editing (Yan et al., 2018). However, more work about the construction of synthetic CRISPR/Cas13d sensing RNA will be performed in the future. Besides, mechanistically, integrated *in silico* analyses and luciferase reporter assays revealed that miR-195/497 cluster directly targeted Yap, SMAD3, BIRC5, and CCND1, the key components in the Hippo and TGF-β pathway. Moreover, we found that miR-195/497 cluster decreased the formation of regulatory transcription complex containing Yap and Smad3, while decreasing their common target genes, such as the gene-encoding CTGF (Luo, 2017). Various studies have revealed that CTGF is critical in cancer progression and initiation (Chen et al., 2019; Pu et al., 2019; Zhou et al., 2020).

Although we focused on the target genes in the Hippo and TGF-β pathway, it is likely that other genes or pathways are changed in BCa cells as a result of miR-195/497 cluster–mediated posttranscriptional gene silencing. For example, miR-195/497 cluster maintained Notch activity and HIF-1α protein expression by targeting FBXW7 in endothelial cells (Yang et al., 2017). However, we observed no similar changes of HIF-1α and FBXW7 expression after miR-195/497 mimic treatment in BCa cells (data was not shown). The reasons for the disparities remain unclear. One possibility is that miRNA performs distinct genetic programs in the different cells and microenvironments in which the cells reside.

Numerous studies points toward an important role for E2F4 with reports of transcriptional stimulatory or repressive effects of E2F4 in different cell type or tissue context (López-Díaz et al., 2013; Cuitiño et al., 2019; Hsu et al., 2019; Kanakkanthara et al., 2019). It is possible that E2F4 serves as a transcriptional activator or repressor upon binding distinct transcriptional cofactors. E2F4 drives genes transcription by interacting with acetyltransferase GCN5 and the essential cofactors TRRAP (Lang et al., 2001). Conversely, E2F4 silence target genes rely on its interaction with the “pocket protein,” such as retinoblastoma (Rb), p107, and p130 that recruit DNA methyltransferases (DNMTs) (Iaquinta and Lees, 2007; López-Díaz et al., 2013). Our data showed that E2F4 represses MIR497HG transcriptional activity, while inhibiting its embedded miR-497 and miR-195 expression. Further studies are needed to investigate whether a novel co-repressor that interacts with E2F4 exists.

In conclusion, E2F4 suppressed the expression of MIR497HG, and MIR497HG suppresses BCa progression through disrupting the crosstalk between Hippo/Yap and TGF-β/Smad signaling by reducing expression of four key genes involved in these two pathways and Yap–Smad3 interaction. Our findings may open up avenues for developing effective therapeutic strategies to treat BCa.

## Data Availability Statement

The original contributions presented in the study are included in the article/Supplementary Materials, further inquiries can be directed to the corresponding author/s.

## Ethics Statement

The studies involving human participants were reviewed and approved by The experimental protocol was established, according to the ethical guidelines of the Helsinki Declaration and was approved by the Human Ethics Committee of the Ethics Committee of Shenzhen University Nanshan Hospital. Written informed consent was obtained from individual or guardian participants. The patients/participants provided their written informed consent to participate in this study.

## Author Contributions

CEZ, CAZ, and YL conceived the project, designed, and performed the research. CAZ, SF, and WY analyzed the data. YL and SF wrote the manuscript. CY, WY, XH, and JL provided assistance in some experiments and reviewing of the manuscript. CEZ and WX provided financial support. All authors contributed to the article and approved the submitted version.

## Conflict of Interest

The authors declare that the research was conducted in the absence of any commercial or financial relationships that could be construed as a potential conflict of interest.

## Supplementary Material

Supplementary Figure 1 Knockdown efficiency of MIR497HG with three diferent siRNA. RT-qPCR assays showed no significant change of MIR497HG in 3 siRNAs transfected cells.

Supplementary Figure 2 MIR497HG suppressed T24 cell growth, migration and invasion *in vitro*. The CCK-8 assay **(A)** and colony formation assays **(B, C)** showed that MIR497HG overexpression repressed T24 cell proliferation. Wound healing assays **(D, E)** and transwell assays **(F, G)** suggested that overexpression of MIR497HG significantly inhibited migratory and invasive activity of T24 cells. Data are shown as mean ± SD. *n* = 3 for technical replicates. ^∗∗^*P* < 0.01 and ^∗∗∗^*P* < 0.001.

Supplementary Figure 3 Expression analysis of selected genes after miR-497 or miR-195 overexpression. Birc5 **(A)**, CCND1 **(B)**, Smad3 **(C),** and Yap **(D)** mRNA levels were detected by RT-qPCR in miR-497 mimic, miR-195 mimic, and mimic-ctrl transfected cells. Data are shown as mean ± SD. *n* = 3 for technical replicates. ^∗∗^*P* < 0.01 and ^∗∗∗^*P* < 0.001.

Supplementary Figure 4 RT-qPCR suggested that the expression of MIR497HG was no changed after knock down of E2F6. ^∗∗∗^*p* < 0.001.

Supplementary Table 1 Sequences for cloning, site directed mutagenesis, ChIP-qPCR and RT-qPCR in this work.

Supplementary Table 2 KEGG pathway enrichment analysis showed that miR-195/497 cluster putative targets involve in different biological pathways.

Supplementary Table 3 The binding of transcription factor and promoter of MIR497HG was predicted by JASPAR database.
