# Corrigendum: Silencing of lncRNA MIR497HG via CRISPR/Cas13d Induces Bladder Cancer Progression Through Promoting the Crosstalk Between Hippo/Yap and TGF-β/Smad Signaling

**DOI:** 10.3389/fmolb.2021.664616

**Published:** 2021-09-23

**Authors:** Changshui Zhuang, Ying Liu, Shengqiang Fu, Chaobo Yuan, Jingwen Luo, Xueting Huang, Weifeng Yang, Wuwei Xie, Chengle Zhuang

**Affiliations:** ^1^ Department of Urology, Union Shenzhen Hospital, Huazhong University of Science and Technology, Shenzhen, China; ^2^ Shenzhen People’s Hospital, The First Affiliated Hospital of Southern University of Science and Technology, The Second Clinical Medical College of Jinan University, Shenzhen, China; ^3^ Department of Urology, The First Affiliated Hospital of Nanchang University, Nanchang, China; ^4^ Emergency Department, Union Shenzhen Hospital, Huazhong University of Science and Technology, Shenzhen, China; ^5^ Shenzhen Yantian District People’s Hospital, Shenzhen, China; ^6^ Department of Thoracic Surgery, Union Shenzhen Hospital, Huazhong University of Science and Technology, Shenzhen, China; ^7^ Department of Urology, Peking University Shenzhen Hospital, Shenzhen, China

**Keywords:** bladder cancer, MIR497HG, CRISPR/Cas13days, YAP, TGF-b

In the original article (Zhuang et al., 2020), there were two errors in Supplementary Figure 2. The “T24 Wound Healing” images of 24 h of “ctrl” in Supplementary Figure 2D and the Supplementary Figure 2F (right) mistakenly used the 5,637 cells data. We then proceed to re-check all original data, found that the inadvertent errors happened during figure processing. All the original pictures of Wound Healing and migration assays of 5,637 and T24 cells were put in a same folder for faster processing and mistakes were happened in the process of image processing using Adobe Illustrator CS6. The authors truly apologize for the oversight on this matter to the editors, reviewers and readers for any confusion that has been caused by these unintentional errors.

The authors apologize for this error and state that this does not change the scientific conclusions of the article in any way. The original article has been updated.

## References

[B1] Zhuang C. Liu Y. Fu S. Yuan C. Luo J. Huang X. (2020). Silencing of lncRNA MIR497HG via CRISPR/Cas13d Induces bladder cancer progression through promoting the Crosstalk between hippo/yap and TGF-β/smad signaling. Front. Mol. Biosci. 7, 616768. 10.3389/fmolb.2020.616768.eCollection2020 33363213 PMC7755977

