# Supplementary figures and images for: Silencing of lncRNA MIR497HG via CRISPR/Cas13d Induces Bladder Cancer Progression Through Promoting the Crosstalk Between Hippo/Yap and TGF-β/Smad Signaling
Source: Front Mol Biosci. 2020 Dec 9;7:616768. doi: 10.3389/fmolb.2020.616768 (PMC7755977; doi:10.3389/fmolb.2020.616768)

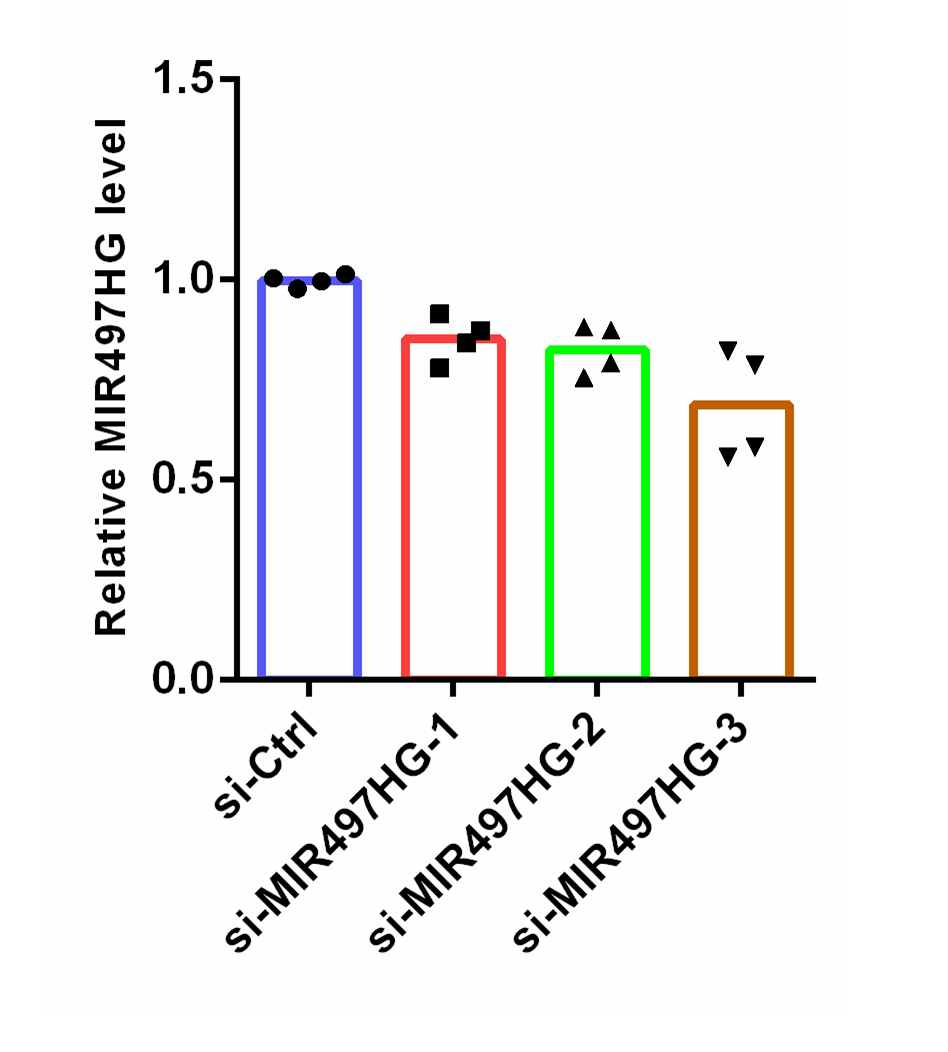

Supplement: Supplementary Figure 1 — Knockdown efficiency of MIR497HG with three diferent siRNA. RT-qPCR assays showed no significant change of MIR497HG in 3 siRNAs transfected cells. [file Image_1.TIF]

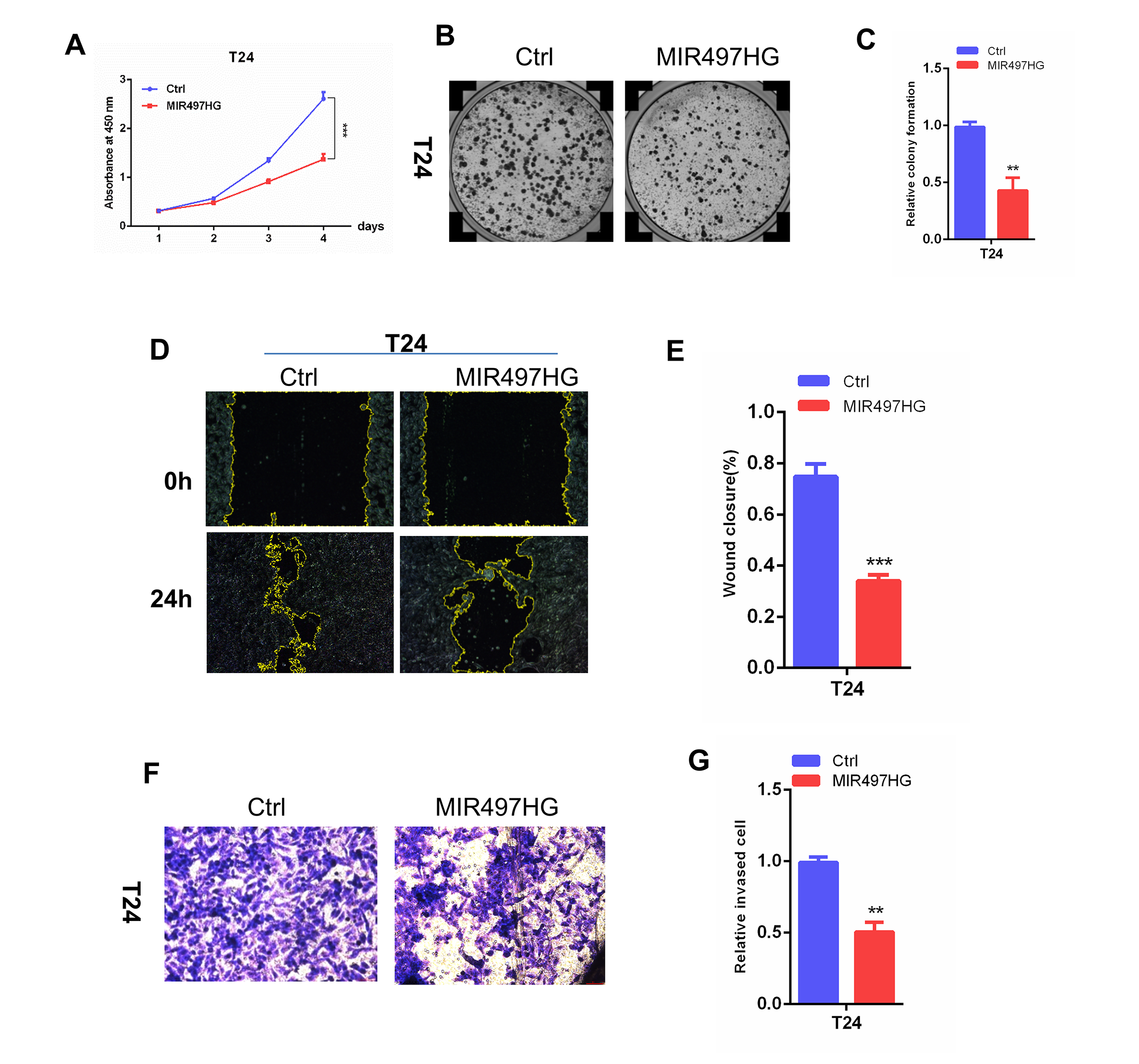

Supplement: Supplementary Figure 2 — MIR497HG suppressed T24 cell growth, migration and invasion in vitro. The CCK-8 assay (A) and colony formation assays (B, C) showed that MIR497HG overexpression repressed T24 cell proliferation. Wound healing assays (D, E) and transwell assays (F, G) suggested that overexpression of MIR497HG significantly inhibited migratory and invasive activity of T24 cells. Data are shown as mean ± SD. n = 3 for technical replicates. ∗∗P < 0.01 and ∗∗∗P < 0.001. [file Image_2.tif]

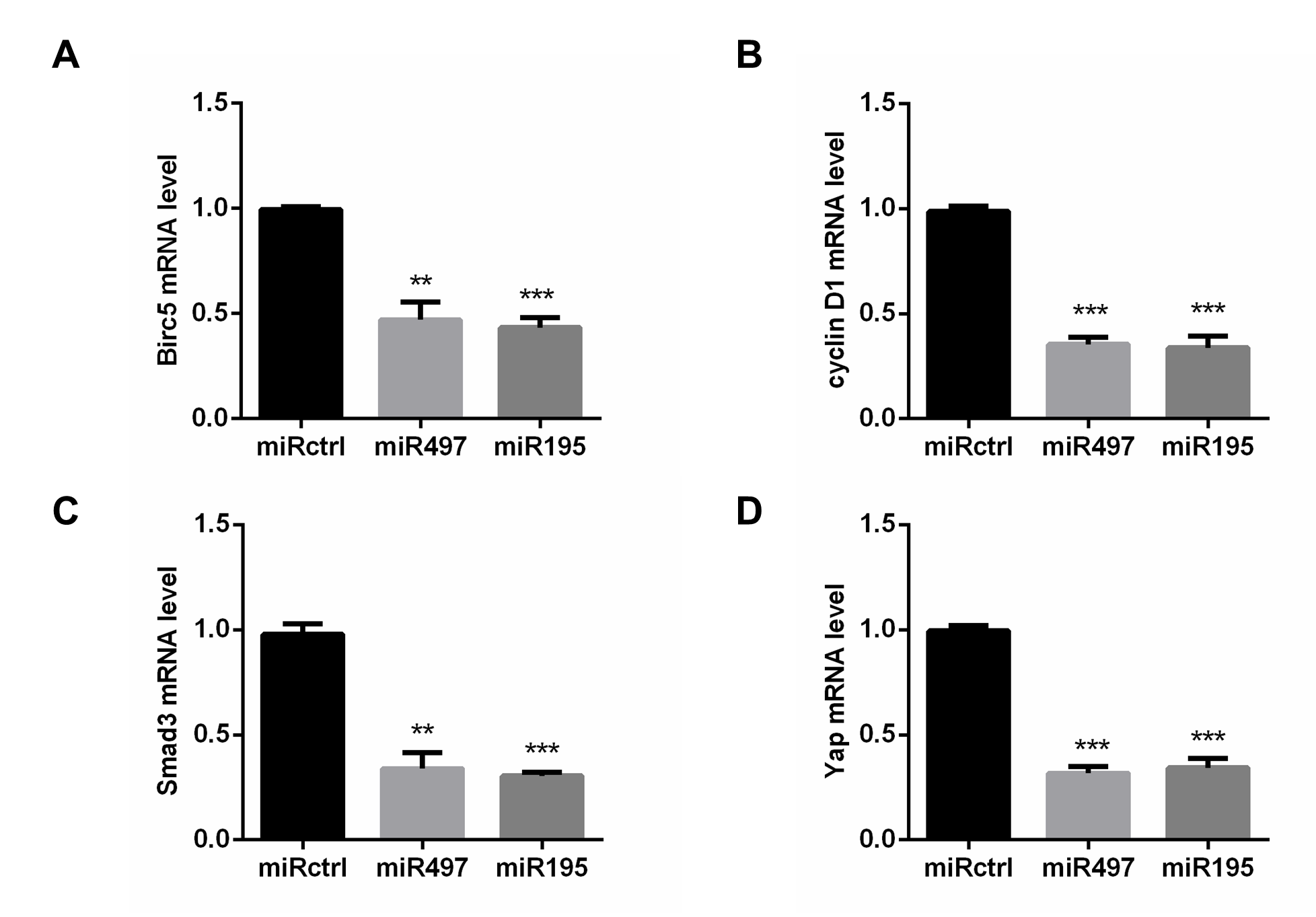

Supplement: Supplementary Figure 3 — Expression analysis of selected genes after miR-497 or miR-195 overexpression. Birc5 (A), CCND1 (B), Smad3 (C), and Yap (D) mRNA levels were detected by RT-qPCR in miR-497 mimic, miR-195 mimic, and mimic-ctrl transfected cells. Data are shown as mean ± SD. n = 3 for technical replicates. ∗∗P < 0.01 and ∗∗∗P < 0.001. [file Image_3.TIF]

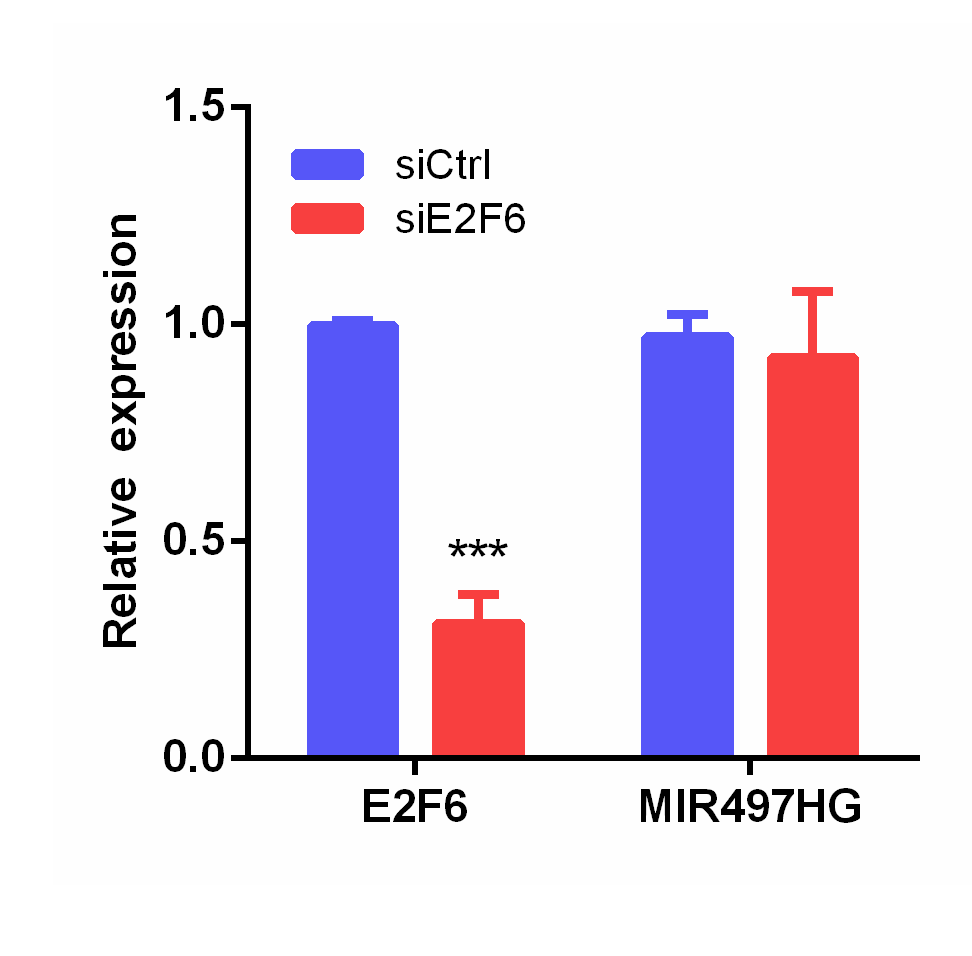

Supplement: Supplementary Figure 4 — RT-qPCR suggested that the expression of MIR497HG was no changed after knock down of E2F6. ∗∗∗p < 0.001. [file Image_4.TIF]
