# Supplementary material for: Silencing of lncRNA MIR497HG via CRISPR/Cas13d Induces Bladder Cancer Progression Through Promoting the Crosstalk Between Hippo/Yap and TGF-β/Smad Signaling
Source: Front Mol Biosci. 2020 Dec 9;7:616768. doi: 10.3389/fmolb.2020.616768 (PMC7755977; doi:10.3389/fmolb.2020.616768)
Supplement: Supplementary Table 1 — Sequences for cloning, site directed mutagenesis, ChIP-qPCR and RT-qPCR in this work. [file Table_1.DOCX]

| Cloning | Symbol | Forward primer (5’-3’) | Reverse primer (5’-3’) |
| --- | --- | --- | --- |
|  | YAP1 | TCGGTCGACATGGATCCCGGGCAGC | GAGAGATCTCTATAACCATGTAAGA |
|  | SMAD3 | CGAGGTACCATGTCGTCCATCCTGCC | CCCGCGGCCGCCTAAGACACACTGGAACA |

| Site directed  mutagenesis | Mutation | Forward primer (5’-3’) | Reverse primer (5’-3’) |
| --- | --- | --- | --- |
|  | MUT1 | ACAGAGCAGGGCTAATAAAAGGCAGGCAAAAGACGGA | TCCGTCTTTTGCCTGCCTTTTATTAGCCCTGCTCTGT |
|  | MUT2 | GCATTGCAGCTGCCTCTTTTATTGAGTTGGAGACGGAT | ATCCGTCTCCAACTCAATAAAAGAGGCAGCTGCAATGC |

| ChIP-qPCR | Symbol | Forward primer (5’-3’) | Reverse primer (5’-3’) |
| --- | --- | --- | --- |
|  | Primer1 | ACTGGGAGTGTGGGTGAGA | GCCGAAGAGAGAAGGGACCC |
|  | Primer2 | GGCATCGGGACAGAGCAGG | AATTTGGGGTCCTCAGATACCC |
|  | Primer3 | GAATCTGACTGGGGCATTGC | CCGTCCCAGCCCATCCGT |

| miRNA | RT-primer sequence |
| --- | --- |
| miR-497 | GGAGAGGAGAGGAAGAGGGAAATCTCCTCTCCACAAAC |
| miR-195 | GTTGGGAGGTAGGAGGTTGATATCCTCCCAACGCCAAT |

| RT-qPCR | Symbol | Forward primer (5’-3’) | Reverse primer (5’-3’) |
| --- | --- | --- | --- |
|  | MIR497HG | CTTACCCAAGGTTCCATCG | CTGCCATCCAGTCCTCAA |
|  | E2F4 | CACCACCAAGTTCGTGTCCC | GCGTACAGCTAGGGTGTCA |
|  | GAPDH | GCATCCTGGGCTACACTGAG | GTCAAAGGTGGAGGAGTGGG |
|  | miR-497 | GCAGCAGCACACTGTG | GAGAGGAGAGGAAGAGGGAA |
|  | miR-195 | AGGGTAGCAGCACAGAAAT | TTGGGAGGTAGGAGGTTGAT |
|  | U6 | CTCGCTTCGGCAGCACA | AACGCTTCACGAATTTGCGT |
